# Intramural Esophageal Hematoma Mimicking Acute Coronary Syndrome: Complication of Warfarin Use in a Mechanical Heart Valve Patient

**DOI:** 10.1155/2022/5636989

**Published:** 2022-06-15

**Authors:** Toktam Alirezaei, Rana Irilouzadian, Ali Pirsalehi, Sayyed Mojtaba Nekooghadam

**Affiliations:** ^1^Cardiology Department of Shohadaye-Tajrish Hospital, Shahid Beheshti University of Medical Sciences, Tehran, Iran; ^2^School of Medicine, Shahid Beheshti University of Medical Sciences, Tehran, Iran; ^3^Department of Internal Medicine, School of Medicine, Shahid Beheshti University of Medical Sciences, Tehran, Iran; ^4^Shohada-Ye-Tajrish Medical Center, Shahid Beheshti University of Medical Sciences, Tehran, Iran

## Abstract

A 59-year-old female with a history of mitral valve replacement presented to emergency department, complaining of sudden-onset retrosternal chest pain since 4 hours ago. Electrocardiogram, laboratory tests, and computed tomography (CT) angiography of aorta were performed and ruled out aortic dissection and cardiovascular events. However, new complaint of odynophagia, dysphagia, and incidental findings in CT angiography proposed esophageal pathologies. After initial workup including upper gastrointestinal endoscopy, intramural esophageal hematoma was diagnosed. Laboratory tests revealed significant reduction in the hemoglobin level. Management of warfarin-induced major bleeding in a patient whom anticoagulation was necessary for the prevention of mechanical heart valve thrombosis was challenging. The patient recovered fully with conservative treatment and was discharged on hospital day 14 with low molecular weight heparin. We described a case of intramural esophageal hematoma as a rare condition that could be misdiagnosed as a cardiovascular emergent disease and would be worsened by antiplatelet and anticoagulation therapy. Accordingly, it is important to differentiate intramural esophageal hematoma from cardiac ischemic events. Another challenge was correction of coagulation in the presence of mechanical mitral valve. Fortunately, we had a favorable outcome following conservative management.

## 1. Introduction

Chest discomfort is certainly one of the most commonly encountered complaints in clinical practice, especially in the emergency setting. Common causes of chest discomfort such as ischemic heart disease, acute aortic syndromes, pulmonary thromboembolism, esophageal problems, and musculoskeletal injuries are familiar for the physicians and are rarely missed by an astute physician after a careful history and physical examination. But in practice, there are some rarer conditions that beside aforementioned diagnoses are usually considered “exceptions” rather than “rules.” Esophageal hematoma is surely one of these “exceptions” that despite its rarity, in the relevant clinical settings (e.g., prior use of anticoagulants and severe thrombocytopenia) should always be sought and ruled out because it obliges the physician to adopt a completely different and sometimes contradictory therapeutic approaches compared with most other causes of chest pain/discomfort [1]. Traumatic or spontaneous intramural hematoma of the esophagus is a rare diagnosis. The most common presenting symptoms include acute chest discomfort/pain, difficulty swallowing, and hematemesis [2]. Seeking for signs and symptoms of this condition is a vital guide to following up these cases and avoiding inappropriate treatment and unnecessary surgical intervention. In this regard, we tried to describe a patient who presented with acute onset chest pain mimicking ischemic heart disease and delineate clinical clues to differentiate it from other diagnoses [3].

## 2. Case Report

A 59-year-old female with a mechanical heart valve, presented to our cardiac emergency department with a severe, sudden onset, compressive retrosternal chest pain, radiating to left hemithorax and back since 4 hours ago. The patient had no prior history of gastrointestinal, pulmonary or bleeding disorders, and endoscopic procedures. She reported no fever, nausea, vomiting, hematemesis, anorexia, or weight loss. Past medical history included hypothyroidism, asthma, and rheumatic heart disease for which she had undergone mitral valve replacement 19 years ago. His daily medications were sodium warfarin (5 mg once daily), acetylsalicylic acid (80 mg once daily), metoprolol, atorvastatin, and levothyroxine. She denied any recent change of warfarin dosage.

Upon admission, vital signs were normal (blood pressure of 135/80 mm·Hg without significant difference between two arms, heart rate of 95 beats per minute, temperature of 36.5°C, respiratory rate of 18 breaths per minute with arterial oxygen saturation of 96% breathing room air).

Physical examination revealed a sternotomy scar. Jugular veins were normal, and cardiac examination showed metallic closing sound from mechanical heart valve prosthesis and a mild right ventricular heave. Pulmonary and abdominal examination were unremarkable. Laboratory tests yielded a hemoglobin concentration of 12.8 g/dL, leukocyte count of 10 × 10^9^/L, platelet count of 197 × 10^9^/L, activated partial thromboplastin time of 30 seconds (normal 26.0–45.0), prothrombin time of 27 seconds (11–13), and international normalized ratio (INR) of 2.52. Other laboratory findings including cardiac and liver enzymes were all within normal limits.

**Figure 1:**
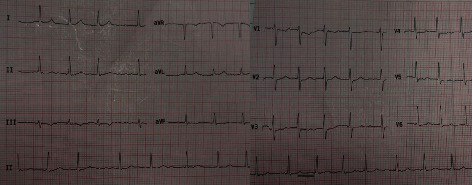
ECG showing atrial fibrillation rhythm.

Chest X-ray was normal. Electrocardiogram (EKG) was obtained and showed atrial fibrillation rhythm with nonspecific ST deviation (Figure 1). Transthoracic echocardiography demonstrated mild left ventricular systolic dysfunction (EF = 45%) with normal hemodynamic and acceptable gradient of mechanical mitral valve prosthesis.

Because of acute retrosternal pain radiating to back, aortic dissection and cardiac ischemic events were our early diagnoses. An EKG inconsistent with ischemia and normal cardiac enzymes ruled out myocardial infarction. The result of computed tomography (CT) angiogram of the aorta ruled out aortic dissection but revealed a mass-like lesion in the margin of the esophagus and posterior mediastinum with an approximate diameter of 52 mm.

**Figure 2:**
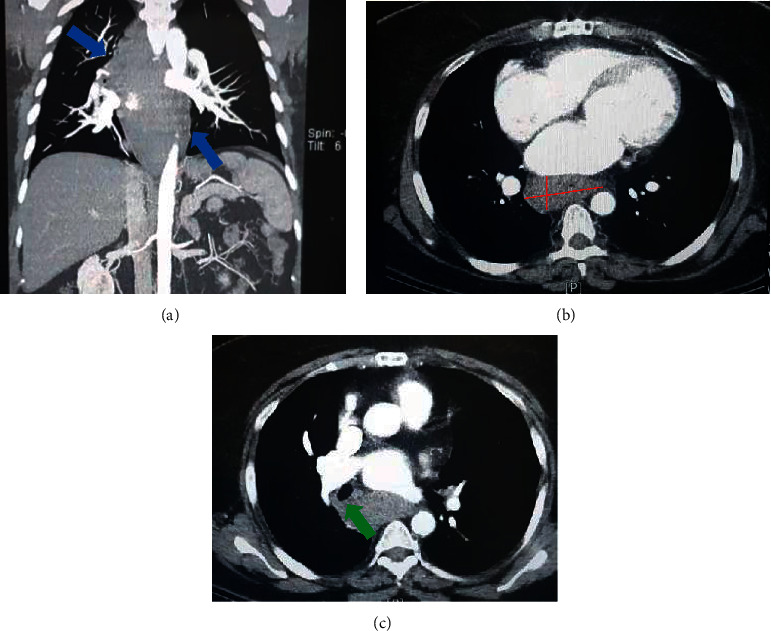
Contrast-enhanced axial and coronal computed tomography of the chest showing an abnormal esophageal contour beginning about 16 cm from incisura extending about 20 cm, terminating on LES ((a), blue arrows). The CT images clearly show esophageal dilatation with a maximum lesion size of 5 × 2.2 cm ((b), red lines), irregularity, and tracheal deviation caused by hematoma ((c), green arrow) *(date performed: December 21, 2021)*.

**Figure 3:**
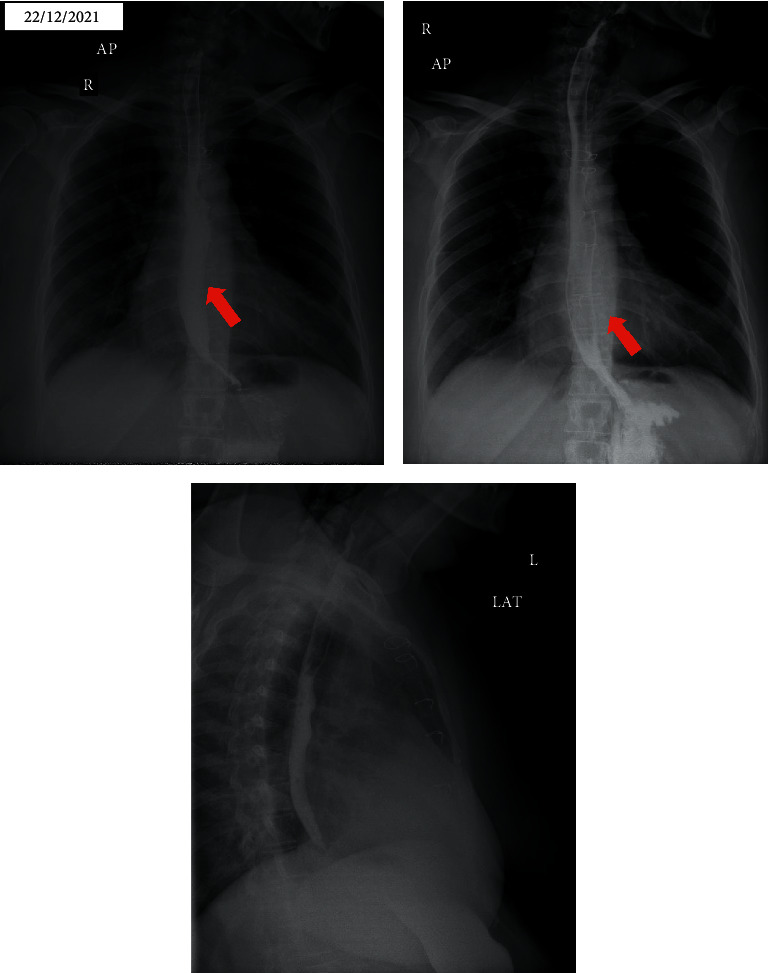
Anteroposterior (AP) and lateral (LAT) chest radiography after barium ingestion showed a mildly dilated and irregular esophageal lumen causing asymmetric wall thickness, especially in anteroposterior projection (red arrows) *(date performed: December 22, 2021)*.

**Figure 4:**
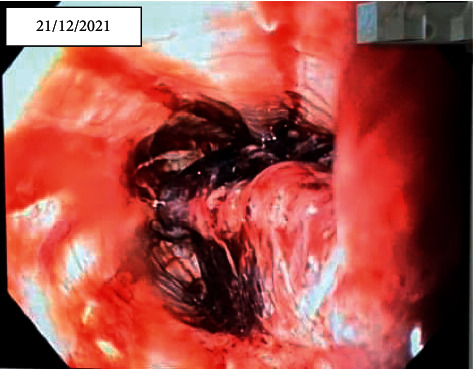
Endoscopic view of esophageal lumen demonstrating extensive mural hemorrhage with clot formation, intraluminal bulging, and significant narrowing *(date performed: December 21, 2021)*.

On the 4th hour of hospital admission, the patient complained of odynophagia, dysphagia, and cold sweating. According to new findings, cardiac ischemic events were improbable, and upper gastrointestinal tract pathologies were considered. Following multidisciplinary consultations, the patient was considered for esophageal imaging studies. The lesion was detected by chest CT scan and upper gastrointestinal tract radiographic study after barium ingestion (Figures 2 and 3). The next step was upper gastrointestinal endoscopy, which demonstrated extensive mural hemorrhage with clot formation in esophageal study (Figure 4).

A conservative medical treatment was chosen. Warfarin and acetylsalicylic acid was discontinued. Intravenous fluid, proton pump inhibitors, and antibiotics were started. The initial hemoglobin concentration was 12.8 g/dL, and 18 hours later, it was 9.2 g/dL. Since the bleeding caused more than 2 g/dL fall in hemoglobin level, it was considered as major bleeding.

The patient had older type of mechanical valve with high thrombogenicity which would markedly increase the rate of valve thrombosis and thromboembolism events. Considering that INR level was in the minimum therapeutic range, it was implausible as a risk factor for bleeding; knowing that anticoagulation therapy was necessary for prevention of mechanical heart valve thrombosis, we were required to reverse anticoagulation in order to stop bleeding. Therefore, management of warfarin-induced major bleeding in this patients was a big challenge for us.

Fresh frozen plasma was administrated, and supportive care was continued. In this setting where anticoagulation was necessary, infusion of heparin with therapeutic dose was started after 48 hours. Dysphagia improved enough to allow a soft diet and fluid; periodic reassessment for symptoms of esophageal hematoma development was performed, and the patient did not have any new complaint. As a result, on hospital day 6, unfractionated heparin was changed to low molecular weight heparin (LMWH) (1 mg/Kg BID). The patient was asymptomatic and hemodynamically stable in the remainder of the hospital stay, tolerating a full diet, and repeated endoscopy showed healing of lesion.

The patient was subsequently discharged on hospital day 14 with a proton pump inhibitor, metoprolol, levothyroxine, and LMWH.

## 3. Discussion

As explained above, in a crowded emergency department, besides common clinical disorders presenting with angina pectoris, some infrequent-and yet important-causes of chest pain require special attention to be diagnosed correctly. One of such misleading diagnoses is esophageal hematoma, which may completely mimic acute coronary syndrome. Intramural esophageal hematoma is a rare esophageal pathology that can occur spontaneously or as a result of a procedure-related complication such as esophageal dilation, variceal band ligation, and endotracheal intubation. The most common predisposing factors include female sex, foreign body ingestion, coagulopathy, and trauma to chest [4–6]. Like our patient, some clinical clues may help clinicians to diagnose it and avoid unnecessary workups. As always, careful history taking and physical examination played the most important role in this scenario. History of consuming antithrombotic drugs, especially anticoagulants such as warfarin, heparin, and novel oral anticoagulants (NOACs), places the patient at a high risk of spontaneous bleeding, including esophageal hematoma [7]. The pain characteristics and accompanying signs and symptoms may also help the physician to differentiate esophageal hematoma from more common causes of acute chest pain. Chest pain with radiation to the arms or jaw is more characteristic of an ischemic chest pain. Diaphoresis is also more commonly seen in coronary patients. Nausea and vomiting may be less helpful in these patients. In contrast, complaints such as dysphagia, odynophagia, or hematemesis are more characteristic for esophageal problems [2]. Lastly, imaging findings including esophageal dilatation in conventional chest radiographs or esophageal wall thickening, irregularities, and luminal narrowing in CT scan images may be very helpful [8]. The gold standard procedure for immediate and correct diagnosis of intramural hematoma is esophageal endoscopy that clearly demonstrates the presence of hematoma, its extension, complications, and may also aid in hematoma aspiration in severe cases to preclude surgical intervention.

Intramural esophageal hematoma is a benign condition which is usually managed by conservative treatment including pain relief, withholding any offending drugs and correcting anemia. As the last line of treatment—if necessary, surgical intervention is required to evacuate the hematoma [9]. However, as it may mimic life-threatening conditions such as myocardial infarction and pulmonary embolism, the misdiagnosis can lead to initiating a contradictory treatment (i.e., thrombolytic and anticoagulation therapy) and, therefore, worsening the condition [10]. A study by Yapa and Green suggested that, in patients presenting with acute chest pain and the absence of electrocardiographic evidence of myocardial infarction, selection of patients for thrombolytic therapy should be done cautiously because of its profound consequences [11].

## Data Availability

The data that support the findings of this study are available on request from the corresponding author.
